# Dietary Trehalose Activates Autophagy Potentially Via MAPK Signaling Pathway to Alleviate Oxidative Stress Induced by Air Exposure Stress in Chinese Mitten Crab (*Eriocheir sinensis*)

**DOI:** 10.1155/anu/5002677

**Published:** 2025-07-03

**Authors:** Anjun Xiang, Jiayi Chen, Jianyang Sun, Chengyi Weng, Yongxu Cheng, Xiaozhen Yang

**Affiliations:** ^1^National Demonstration Center for Experimental Fisheries Science Education, Shanghai Ocean University, Shanghai 201306, China; ^2^Key Laboratory of Freshwater Aquatic Genetic Resources, Ministry of Agriculture and Rural Affairs, Shanghai Ocean University, Shanghai 201306, China; ^3^Shanghai Engineering Research Center of Aquaculture, Shanghai Ocean University, Shanghai 201306, China; ^4^Key Laboratory of Integrated Rice-Fish Farming Ecosystem, Ministry of Agriculture and Rural Affairs, Shanghai Ocean University, Shanghai 201306, China

**Keywords:** air exposure stress, autophagy, *Eriocheir sinensis*, oxidative stress, trehalose

## Abstract

Air exposure stress (AES) is a common stress faced by Chinese mitten crabs (*Eriocheir sinensis*) in aquaculture and transport. This study was designed to evaluate the levels of endogenous trehalose, oxidative stress, and autophagy in crabs subjected to AES (0, 24, and 48 h) after 14 days of feeding diets with different levels of trehalose (Diet1, 0 g/kg; Diet2, 1 g/kg; Diet3, 5 g/kg; and Diet4, 10 g/kg). The results showed that after AES, the endogenous trehalose in crabs was significantly reduced, the expression of trehalose hydrolase (*TREH*) gene were significantly downregulated, while trehalose-6-phosphate synthase (*TPS*) gene and trehalose transporters (*TRET*) gene were remarkably upregulated. These results showed that crabs exhibited an increased demand for trehalose under AES. AES induced oxidative stress damage in the hepatopancreas of crabs resulting in hepatic tubules atrophy, increased vacuolated cells, significantly increased catalase (CAT) activity and malondialdehyde (MDA) content, and decreased superoxide dismutase (SOD) activity. Dietary trehalose supplementation significantly upregulated the expression of *TREH* gene in crabs, significantly downregulated the expression of *TPS* gene, and notably enhanced trehalose content. Dietary supplementation with trehalose mitigated hepatopancreatic oxidative damage in crabs subjected to AES. At 48 h of AES, the hepatopancreas of the Diet4 group maintained histological normality, with significantly decreased CAT activity and MDA content, and significantly increased SOD activity. Notably, the protective effect of trehalose may be attributed to its activation of autophagy. Dietary trehalose supplementation significantly upregulated the expression of autophagy-related genes (*ATG4B*, *ATG7*, *ATG13*, and *Beclin1*) and canonical autophagic MAPK signaling cascade (*ERK*, *JNK*, and *p38*), with p38 protein results further indicating potential activation of this pathway. These findings indicated that dietary trehalose supplementation influences the regulation of endogenous trehalose in crabs, potentially modulating autophagy via the MAPK signaling pathway to alleviate oxidative stress induced by AES.

## 1. Introduction

Chinese mitten crab (*Eriocheir sinensis*) is one of the critical economic species in China's aquaculture industry [1]. During aquaculture and transportation, crabs are highly susceptible to air exposure stress (AES) [2, 3]. AES, defined as the physiological stress imposed on aquatic animals when removed from water, can occur during short-term or prolonged water deprivation [4]. Numerous studies have demonstrated that AES induces oxidative stress in aquatic animals, characterized by dysregulated antioxidant enzymes and histopathological damage in the hepatopancreas. However, investigations into AES in crabs remain poorly understood, and further research in this regard could offer valuable theoretical perspectives for the cultivation and transportation of crabs.

Trehalose, a nonreducing disaccharide (α-D-glucopyranosyl-α-D-glucopyranoside), is ubiquitously distributed in organisms such as insects, plants, and crustaceans [5–7]. In insects, trehalose metabolism is well characterized: *TPS* catalyzes trehalose synthesis in the fat body, while *TRET* mediates its transport into hemolymph [8]. *TREH* further regulates hydrolysis of trehalose to glucose, with its activity modulated by environmental stress and dietary intake [9–11]. However, trehalose metabolic pathways in crabs remain poorly understood. Despite *TPS* and *TRET* being conserved across arthropods, their specific roles in crab hepatopancreas or stress responses have not been characterized. Moreover, whether dietary trehalose supplementation or AES modulates trehalose metabolism in crabs remains entirely unknown. Research on trehalose in crustaceans could pave the way for evidence-based trehalose supplementation strategies in crustacean farming.

Autophagy is an evolutionarily conserved catabolic process in eukaryotes, regulated by autophagy-related genes (*ATGs*), which functions by sequestering misfolded protein aggregates and damaged organelles into double-membrane autophagosomes for subsequent lysosomal degradation [12, 13]. In normal conditions, the level of autophagy in cells remains extremely low [14]. However, upon exposure to stressors such as nutrient starvation, environmental stress, and oxidative stress, autophagic flux is robustly induced via conserved signaling pathways [15, 16]. Emerging evidence has indicated that enhanced autophagy alleviates oxidative stress damage and confers protection in various organisms [17, 18]. Among autophagic process, the mitogen-activated protein kinase (MAPK) signaling pathway has been implicated in autophagy regulation [19]. It has been found that trehalose can activate mammalian autophagy through the MAPK signaling pathway. Limited research has been conducted on autophagy in crabs, and it remains uncertain whether trehalose can promote autophagy and mitigate oxidative stress in crabs.

To mitigate the economic losses caused by AES in aquaculture and transportation, feed additives have emerged as a critical and nutritionally manipulable strategy to enhance stress tolerance in aquatic animals [20]. However, limited research has addressed how supplementation in feed modulates antioxidant responses and autophagic regulation in crabs exposed to AES. Therefore, this study aimed to investigate the antioxidant effect in crabs under AES and evaluate the protective mechanisms of trehalose in mitigating oxidative damage and regulating autophagy in crabs during AES.

## 2. Materials and Methods

### 2.1. Preparation of Experimental Diets

The composition and proportions of the basal diets are shown in Table 1 [21]. The ingredients were ground into a fine powder and passed through a 187.5 µm sieve, and trehalose (purity ≥99.0%, Shanghai Yuan Ye, China) was added to each group of fine powders at a concentration of 0 g/kg (Diet 1), 1 g/kg (Diet 2), 5 g/kg (Diet 3), and 10 g/kg (Diet 4), which was then mixed in a household dough mixer (Jiuyang, China). It was then dried in an oven at 65°C. After cooling under natural conditions, it was stored in a refrigerator at −20°C.

### 2.2. Design of Experiments

All juvenile crabs (Shanghai Xinjian, China) were selected and purchased about 15 g. Juvenile crabs were temporarily acclimated in the recirculating water system for 3 days. At the end of acclimation, 240 crabs with healthy bodies, neat specification, and intact appendages, with an average body weight about 15 g, were randomly divided into four groups: Diet 1 (control group), Diet 2, Diet 3, and Diet 4 groups, each with three parallel tanks (20 crabs per tank) and a total of 12 tanks (the tanks were recirculating aquaculture systems, with the water maintained at 24°C, total ammonia nitrogen <0.3 mg/L, pH = 8.0 ± 0.2, dissolved oxygen >5 mg/L, and continuous oxygen supply for 24 h). The group fed the experimental crabs daily (at 6:00 p.m.) at the rate of 3% of the crab's original body weight for a period of 14 d.

After the 14 d of the culture experiment, 30 crabs were randomly selected from tanks in each of the four groups, respectively, and then divided into three parallel tanks for AES (0, 24, and 48 h) (a total of 12 anhydrous drying tanks, room temperature maintained at 24–25°C, and air humidity at 65%) [22].

### 2.3. Sample Collecting

**Figure 1:**
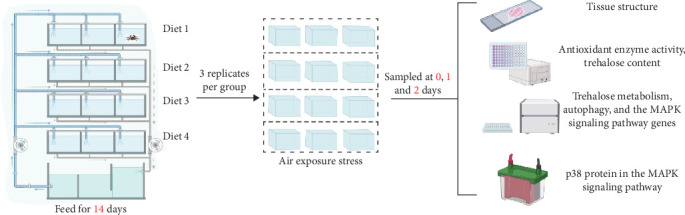
Schematic diagram of the experimental process.

Five crabs from each group were randomly selected for sampling at each period. Hemolymph samples were collected using a syringe and stored in a refrigerator at 4°C for trehalose content testing. Hepatopancreas samples were collected and fixed in Bouin's solution for histological analysis; the remaining samples were stored in a fridge at −80°C for the detection of trehalose content and antioxidant enzyme activities, as well as for further investigations into the gene expression related to metabolism of trehalose, autophagy, and the MAPK signaling pathways. The experimental procedure is outlined in Figure 1.

### 2.4. Analysis

#### 2.4.1. Trehalose Content and Antioxidant Enzyme Activity

The trehalose content in hepatopancreas and hemolymph was determined by the kit (Nanjing Jiancheng, China), and the activities of total antioxidant capacity (T-AOC), superoxide dismutase (SOD), glutathione peroxidase (GSH-PX), catalase (CAT), and the content activity of malondialdehyde (MDA) were similarly detected by the kit [23]. The assays were carried out at 620 nm (trehalose), 520 nm (T-AOC), 550 nm (SOD), 412 nm (GSH-PX), 405 nm (CAT), and 660 nm (MDA) by UV spectrophotometer (Beijing Pukenjie, China). Refer to manufacturer's instruction manual for details.

#### 2.4.2. Histological Analysis

Hepatopancreas samples were taken from each group, cut into pieces of ~5 mm × 5 mm in size, and fixed in Bouin's solution for at least 24 h. Then fixed hepatopancreas was trimmed into blocks, placed in embedding boxes, dehydrated in a graded ethanol solution, xylene removed, paraffin embedded, and made into 4 µm sections, which were then stained with hematoxylin and eosin. The sections were examined using an Olympus BH-2 microscope.

#### 2.4.3. Trehalose Metabolism, Autophagy, and MAPK Signaling Pathway-Related Genes Expression and Real-Time PCR Analysis

Total RNA was extracted from each set of hepatopancreas samples using the RNAiso Plus kit (Takara, China) following the manufacturer's instruction manual. The concentration and quality of the RNA were evaluated through absorbance measurements (OD 260/280). High-quality RNA samples (OD 260/280 range 1.8–2.0) were screened for reverse transcription to synthesize cDNA using the PrimeScript RT Reagent Kit and the gDNA Eraser (Takara, China) and stored in a −20°C refrigerator for backup. RT-PCR was performed using SYBR1 Premix Ex Taq (Takara, China) to quantify the expression levels of genes in the hepatopancreas related to trehalose metabolism, autophagy, and MAPK signaling pathway. Specific primers were designed for RT-qPCR (Table 2). Gene expression levels were quantified using the 2^−*ΔΔ*CT^ method, using the 18S gene as an internal reference.

#### 2.4.4. Western Blot Detection of p38

Hepatopancreas samples were taken from each group and used in RIPA lysis buffer (Sangong, Shanghai, China). After sonication, the resulting supernatant was then collected, and the protein concentration was assessed using the BCA kit (Nanjing Jiancheng, China). The supernatant was then mixed with SDS-PAGE protein upload buffer (Biosharp, China) and boiled. Proteins were separated by 12.5% SDS-PAGE gels (upper gel: 80 V, 30 min; lower gel: 85 V, 65 min) and transferred to PVDF (80 V, 65 min, Biosharp, Shanghai, China). The PVDF membranes were incubated in a blocking buffer (5% skimmed milk powder, 20 mmol L^−1^ Tris–HCl, 150 mmol L^−1^ NaCl, 0.05% Tween-20) at room temp`erature for 2 h. Subsequently, the membranes were incubated with the p38 antibody (1:3000) and β-actin antibody (1:3000) (all from Solarbio, Beijing, China) at 4°C overnight. The membrane was washed three times with TBST solution (20 mmol L^−1^ Tris–HCl, 150 mmol L^−1^ NaCl, 0.05% Tween-20), with each wash lasting 8 min. Then, the membrane was incubated with a secondary antibody (goat anti-mouse IgG, 1:5000, Sangong, Shanghai, China) for 2 h at room temperature. After that, the membrane was washed with TBST solution 3 times (10 min each). The membranes were incubated in High Sensitivity Plus ECL Luminescent Reagent (Sangon, Shanghai, China), and the intensity of the bands was quantified using ImageJ software after exposure to X-OMAT film (Eastman Kodak, USA).

### 2.5. Statistical Analysis

The SPSS 21 statistical software (USA; 21.0) was used to determine statistical differences with one-way analysis of variance (ANOVA) and Duncan's multiple range tests. Results are expressed as mean ± SD. Different lowercase letters are used for same diets at the various time point to indicate statistical significance (*p* < 0.05). Different uppercase letters are used for different diets at the same time point to indicate statistical significance (*p* < 0.05).

## 3. Results

### 3.1. Trehalose Metabolism

#### 3.1.1. Trehalose Content

**Figure 2:**
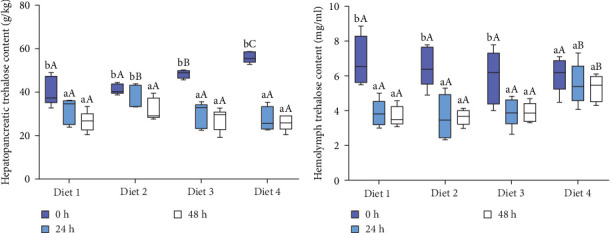
Effect of dietary trehalose on trehalose content in hepatopancreas (A) and hemolymph (B) of crabs exposed to AES. Results are expressed as mean ± SD. (*n* = 5). Different lowercase letters are used for same diets at the various time point to indicate statistical significance (*p* < 0.05). Different uppercase letters are used for different diets at the same time point to indicate statistical significance (*p* < 0.05).

Before AES (0 h), hepatopancreatic trehalose levels exhibited a dietary concentration-dependent increase, with the Diet 4 group showing significantly higher content than other groups (*p* < 0.05; Figure 2A). The content trehalose of the hepatopancreas in all groups significantly declined with extended AES (*p* < 0.05; Figure 2A).

As AES duration increased, the trehalose levels in the hemolymph of all groups exhibited a downward trend, reaching their lowest point at 48 h of AES (*p* < 0.05; Figure 2B). At 24 and 48 h of AES, the hemolymph trehalose content was significantly higher in the Diet 4 group compared to the Diet 1, Diet 2, and Diet 3 groups (*p* < 0.05; Figure 2B).

#### 3.1.2. Expression of Trehalose Metabolism

**Figure 3:**
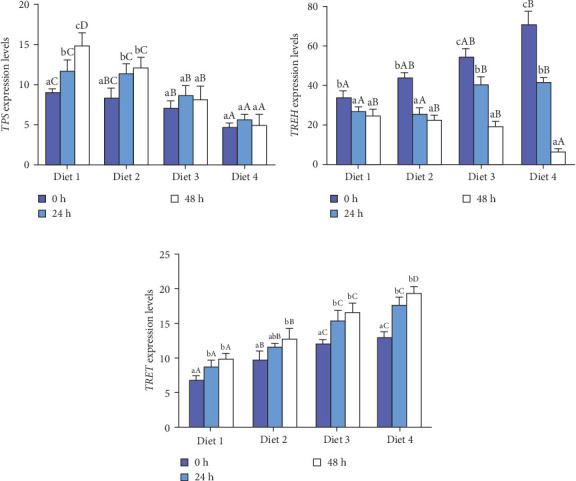
Effect of dietary trehalose on gene expression of trehalose-6-phosphate synthase (A, *TPS*), trehalose (B, *TREH*), and trehalose transporter protein (C, *TRET*) in crabs exposed to AES. Results are expressed as mean ± SD. (*n* = 5). Different lowercase letters are used for same diets at the various time point to indicate statistical significance (*p* < 0.05). Different uppercase letters are used for different diets at the same time point to indicate statistical significance (*p* < 0.05).

As AES duration increased, the expression of *TPS* in the Diet 1 and Diet 2 groups increased significantly (*p* < 0.05; Figure 3A). In contrast, the expression of *TPS* in the Diet 3 and Diet 4 groups showed a trend of first increasing and then decreasing. With the increase in trehalose content in the feed, the expression of *TPS* in all groups exhibited a downward trend (*p* < 0.05; Figure 3A).

As AES duration prolonged, the expression of *TREH* in all groups exhibited a significant downward trend (*p* < 0.05; Figure 3B). At 0 and 24 h of AES, the expression of *TREH* showed a significantly upward trend with the increase of dietary trehalose (*p* < 0.05; Figure 3B). At 48 h of AES, the expression of *TREH* in the Diet 4 group was significantly downregulated in comparison with the other experimental groups (*p* < 0.05; Figure 3B).

At 0, 24, and 48 h of AES, the expression of *TRET* tended to ascend with the increase of dietary trehalose (*p* < 0.05; Figure 3C), and the expression of *TRET* in the Diet 4 group was significantly higher than that in the remaining experimental groups (*p* < 0.05; Figure 3C). Moreover, the expression of *TRET* in all groups gradually increased with longer durations of AES (*p* < 0.05; Figure 3C).

### 3.2. Antioxidant Function

#### 3.2.1. Histological Structure of Hepatopancreas

**Figure 4:**
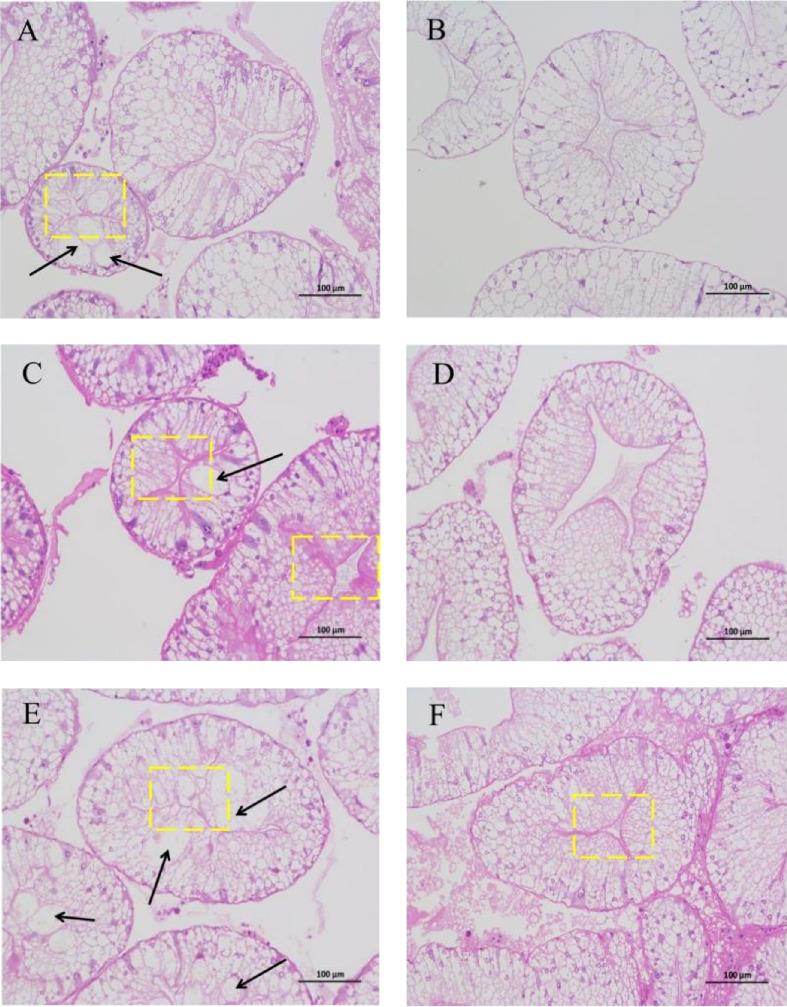
Effect of dietary trehalose on the histological structure of hepatopancreas in crabs exposed to AES. (*n* = 3). Diet1 group, 0 h (A); Diet4 group, 0 h (B); Diet1 group, 24 h (C); Diet4 group, 24 h (D); Diet1 group, 48 h (E); Diet4 group, 48 h (F). The yellow boxes indicate atrophied lumen. The black arrows indicate cell vacuolization.

Before AES (0 h), the hepatopancreas of the Diet 1 group exhibited partial vacuolated cells and focal lumen atrophy, whereas the Diet 4 group displayed hepatopancreas cells with orderly arrangement and no evident vacuolation (Figure 4A, B). AES (24 and 48 h) induced severe hepatopancreatic damage in Diet 1, characterized by lumen atrophy, vacuolar degeneration of epithelial cells, and disorganized architecture (Figure 4C, E). In contrast, Diet 4 maintained near-normal histology at 24 and 48 h, with minimal vacuolization and preserved tubule integrity (Figure 4D, F).

#### 3.2.2. Antioxidant Enzyme Activity

**Figure 5:**
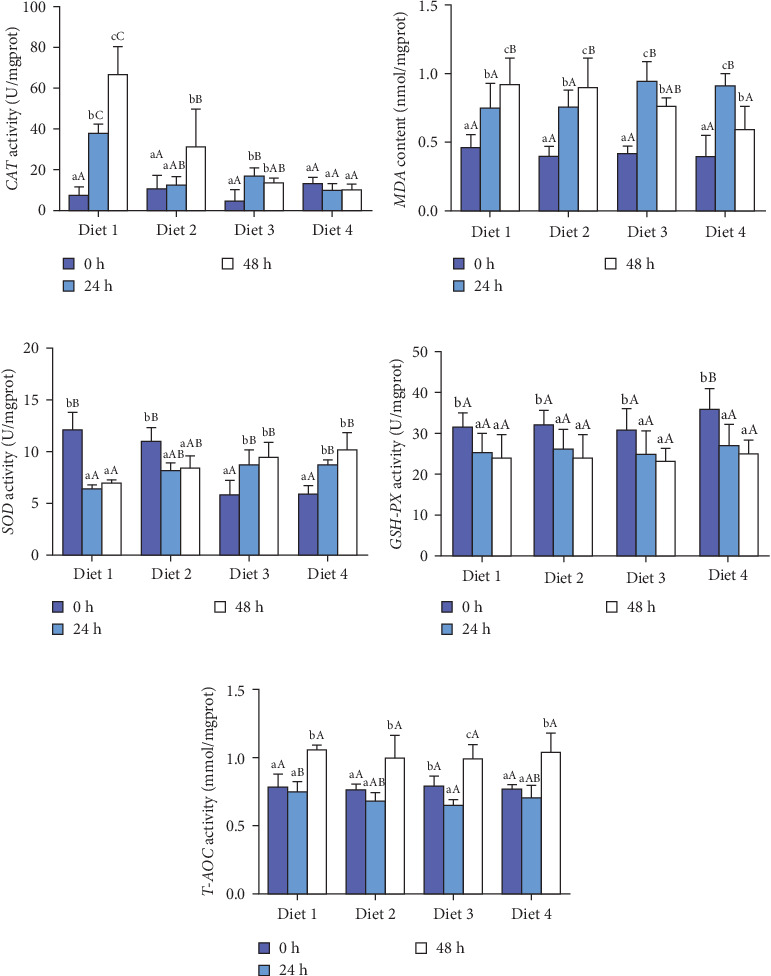
Effects of dietary trehalose on antioxidant enzyme activities in crabs exposed to AES. Catalase (A, CAT), malondialdehyde (B, MDA), superoxide dismutase (C, SOD), glutathione peroxidase (D, GSH-PX), and total antioxidant capacity (E, T-AOC). Results are expressed as mean ± SD. (*n* = 5). Different lowercase letters are used for same diets at the various time points to indicate statistical significance (*p* < 0.05). Different uppercase letters are used for different diets at the same time point to indicate statistical significance (*p* < 0.05).

Before AES (0 h), GSH-PX activity in the Diet 4 group was significantly higher than that in the Diet 1 group (*p* < 0.05; Figure 5D). Conversely, SOD activity in the Diet 3 and Diet 4 groups was significantly lower than that in the Diet 1 and Diet 2 groups (*p* < 0.05; Figure 5C). No significant differences were observed in T-AOC, CAT activity, and MDA content (*p* > 0.05; Figure 5A, B, E).

At 24 h of AES, there were no significant differences in GSH-PX activity across all groups (*p* > 0.05; Figure 5D). Compared with the Diet 1 group, the Diet 4 group exhibited significantly higher SOD activity and MDA content, accompanied by significantly lower CAT activity (*p* < 0.05; Figure 5A).

At 48 h of AES, CAT activity and MDA content in the Diet 4 group were significantly lower than those in the Diet 1 and Diet 2 groups (*p* < 0.05; Figure 5A, B), while SOD activity was significantly higher (*p* < 0.05; Figure 5C). No significant differences in GSH-PX and T-AOC activity were detected between the Diet 1 and Diet 2 groups at 48 h of AES (*p* > 0.05; Figure 5D, E).

### 3.3. Activation and Regulation of Autophagy

#### 3.3.1. Expression of Autophagy Genes

**Figure 6:**
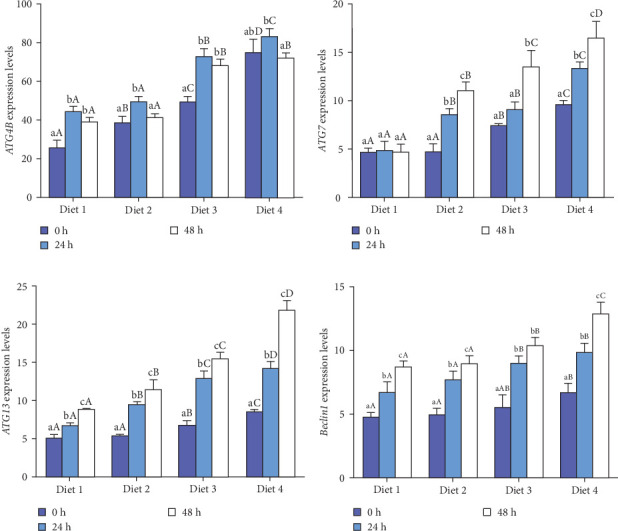
Effect of dietary trehalose on the expression of autophagy-related genes in crabs exposed to AES. Autophagy-related protease 4B (A, *ATG4B*), autophagy-related protein 7 (B, *ATG7*), autophagy-related protein 13 (C, *ATG13*), and BECN 1 (D, *Beclin1*). Results are expressed as mean ± SD. (*n* = 5). Different lowercase letters are used for same diets at the various time point to indicate statistical significance (*p* < 0.05). Different uppercase letters are used for different diets at the same time point to indicate statistical significance (*p* < 0.05).

At each time point, the expression of autophagy-related genes (*ATG4B*, *ATG7*, *ATG13*, *Beclin1*) displayed a dietary concentration-dependent upregulation (*p* < 0.05; Figure 6A–D). Specifically, the expression of f autophagy-related genes (*ATG4B*, *ATG7*, *ATG13*, *Beclin1*) in the Diet 4 group (10 g/kg trehalose) was significantly higher than the control group (Diet 1, 0 g/kg trehalose) (*p* < 0.05; Figure 6A–D).

For *ATG4B*, the expression peaked at 24 h of AES and then downregulated with AES duration increased (*p* < 0.05; Figure 6A). In contrast, the expression of *ATG13* and *Beclin1* showed a gradual, sustained upregulation throughout the extended AES duration (*p* < 0.05; Figure 6C, D).

Notably, the expression levels of *ATG7* in the Diet 1 group was significantly higher than in the other experimental groups (*p* < 0.05; Figure 6B). With the prolongation of AES, no significant difference was observed in the Diet 1 group (*p* > 0.05; Figure 6B), while the expression of *ATG7* in the other experimental groups upregulated significantly with stress time (*p* < 0.05; Figure 6B).

#### 3.3.2. Expression of MAPK Signaling Pathway-Related Genes

**Figure 7:**
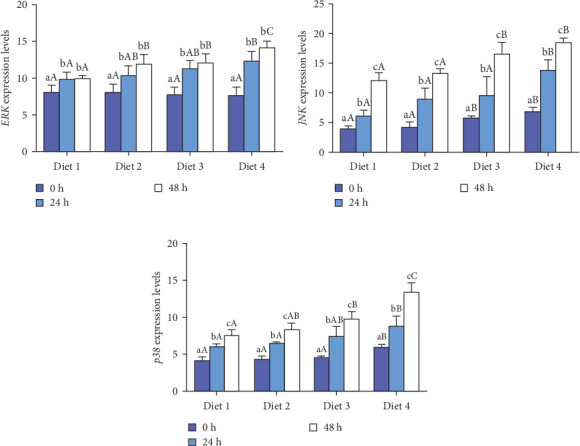
Effect of dietary trehalose on the expression of genes related to MAPK signaling pathway in crabs exposed to AES. Extracellular regulated protein kinase (A, *ERK*), C-Jun N-terminal kinase (B, *JNK*), p38 mitogen-activated protein kinase (C, *p38*). Results are expressed as mean ± SD. (*n* = 5). Different lowercase letters are used for same diets at the various time point to indicate statistical significance (*p* < 0.05). Different uppercase letters are used for different diets at the same time point to indicate statistical significance (*p* < 0.05).

Before AES (0 h), no statistically significant difference was observed in the expression of *ERK* (*p* > 0.05; Figure 7A), while the expression of *JNK* and showed a significant upregulation with the rising concentration of dietary trehalose (*p* < 0.05; Figure 7B, C). The expression of *ERK*, *JNK*, and *p38* was significantly upregulated over time (*p* < 0.05; Figure 7A–C). At the 24 and 48 h of AES, the expression of *ERK*, *JNK*, and *p38* in the Diet 4 group was markedly higher than those in the other experimental groups (*p* < 0.05; Figure 7B, C).

#### 3.3.3. p38 Protein Levels

**Figure 8:**
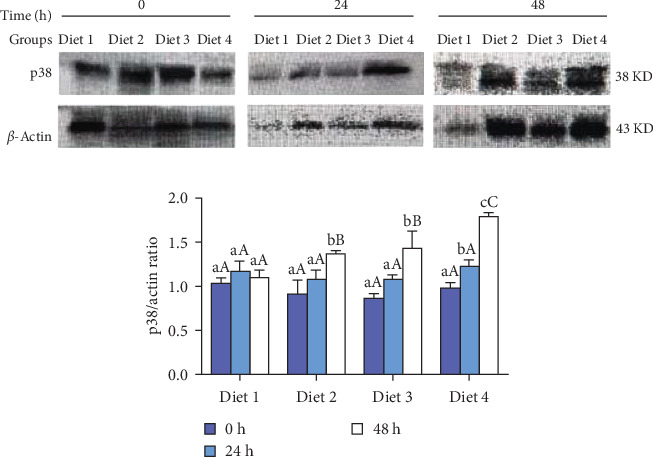
Effect of trehalose on p38 protein levels in crabs exposed to AES (A) and quantified by ImageJ (B). Results are expressed as mean ± SD. (*n* = 3). Different lowercase letters are used for same diets at the various time point to indicate statistical significance (*p* < 0.05). Different uppercase letters are used for different diets at the same time point to indicate statistical significance (*p* < 0.05).

Before AES (0 h), no significant differences in p38 protein levels among the groups were demonstrated (*p* > 0.05; Figure 8B). As the duration of AES was extended, the p38 protein levels of Diet 2, Diet 3, and Diet 4 groups gradually increased (*p＜*0.05; Figure 8B). At 24 h of AES, no significant differences in p38 protein levels were detected among the groups (*p* > 0.05; Figure 8B). At 48 h of AES, the p38 protein levels showed a dietary concentration-trehalose variation, with the Diet 4 group exhibiting a significantly higher level of p38 protein than the other experimental groups (*p* < 0.05; Figure 8B).

## 4. Discussion

### 4.1. Endogenous Trehalose Regulation

Trehalose is widely distributed in plants and lower animals, such as pseudocoelomates, insects, and crustaceans [24, 25]. Numerous studies have demonstrated that regulating endogenous trehalose serves as a critical pathway for the organism to cope with external stresses [26]. In general, an elevation of the organism's trehalose content helps it to acclimate to environmental stresses, though significant differences exist among species [27]. In this study, AES was found to reduce trehalose content in *E. sinensis*, contrasting with the increase observed in the pine wood nematode (*Bursaphelenchus xylophilus*) and muscle larvae (*Trichinella spiralis*) [28, 29], but aligning with the decrease seen in quinoa leaves (*Chenopodium quinoa*) under salinity stress [30]. These discrepancies likely reflect species-specific adaptive strategies to different stress types (e.g., osmotic vs., oxidative stress).

In response to AES-induced trehalose depletion, crabs attempted to compensate by upregulating trehalose-6-phosphate synthase (*TPS*) gene expression anting trehalose hydrolase (*TREH*) gene expression, similar to metabolic adjustments observed in the oriental fruit fly (*Bactrocera dorsalis*) [31]. Notably, exogenous trehalose intake significantly downregulated *TPS* and upregulated *TREH* expression, suggesting dietary trehalose may inhibit its synthesis pathway via negative feedback while relying on exogenous sources for homeostasis. Additionally, trehalose transporter (*TRET*) gene expression positively correlated with dietary trehalose concentration (Figure 3C), consistent with the role of *TRET* in mediating trans-membrane trehalose transport in *Xenopus laevis* oocytes [32], indicating that *TRET* may play a critical role in maintaining hemolymph trehalose levels.

### 4.2. Tissue Structure and Antioxidant Enzyme Activity

The evaluation of hepatopancreatic tissue structure is frequently employed as an experimental approach to assess the extent of oxidative stress in crustaceans [33]. This study revealed that AES caused severe histological damage in control crabs, characterized by lumen atrophy, vacuolar degeneration of epithelial cells, and disorganized architecture, consistent with findings in the ridge-tailed white shrimp (*Palaemon carinicauda*) [34]. In contrast, crabs supplemented with exogenous trehalose maintained nearly normal hepatopancreatic histology at 24 and 48 h of AES, with minimal vacuolization (Figure 4D, F), indicating trehalose alleviates AES-induced tissue injury.

Oxidative stress is one of the main focuses of AES studies, especially in crustaceans [35, 36]. AES was found to disrupt the antioxidant defense system in crabs, evidenced by increased MDA content and decreased SOD activity, consistent with physiological responses in mud crabs (*Scylla paramamosain*) [37]. Notably, dietary trehalose significantly enhanced SOD activity and reduced CAT activity and MDA content under AES (Figure 5A–C), mirroring results in mice [38] and peach fruits [39], which suggests trehalose alleviates oxidative damage by enhancing antioxidant enzyme coordination.

An intriguing observation was the significantly lower SOD activity in the high-trehalose group (Diet4) at pre-stress (0 h) compared to the control group (Figure 5C). This may reflect bidirectional regulation of basal antioxidant enzyme levels by trehalose. A plausible explanation is that high-concentration trehalose activates basal autophagy to promote clearance of damaged mitochondria, thereby reducing superoxide anion production and inducing compensatory decreases in SOD activity [40]. Concurrently, GSH-PX activity was significantly elevated in this group (Figure 5D), suggesting trehalose preferentially enhances the basal activity of nonenzymatic antioxidant systems (e.g., glutathione pathways) to maintain redox balance. The link between these basal enzyme changes and autophagy activation lies in autophagy's role in degrading oxidatively damaged proteins and organelles, reducing the consumption pressure on antioxidant enzymes and optimizing the overall efficiency of the antioxidant defense system [18, 41].

### 4.3. Autophagy and MAPK Signaling Pathways

Environmental stresses often activate autophagy, a conserved catabolic process in eukaryotes that degrades cytoplasmic proteins to protect cells [42, 43]. This study found that AES activated autophagy in crabs, as evidenced by upregulated expression of autophagy-related genes (*ATG4B*, *ATG7*, *ATG13*, *Beclin1*), consistent with findings in the white shrimp (*Penaeus vannamei*) [44, 45]. As a common autophagy activator, trehalose stimulates autophagic processes in response to oxidative stress [46, 47]. Although similar results have been reported in mice [38], research in crustaceans remains limited. This study is the first to demonstrate that dietary trehalose activates autophagy and mitigates AES-induced oxidative damage in crab hepatopancreas.

Autophagy is regulated by multiple signaling pathways, including the mTOR and MAPK pathways [3, 48]. The MAPK pathway is essential for autophagy regulation, and trehalose has been shown to activate autophagy via this pathway [49, 50]. In this study, dietary trehalose significantly upregulated the expression of *ERK*, *JNK*, and *p38* genes under AES, with p38 protein levels showing consistent trends with mRNA expression (Figure 7C). As a key member of the MAPK family, p38 initiates autophagy by phosphorylating downstream targets and upregulating autophagy-related genes (e.g., *ATG7*, *Beclin1*) [51], a mechanism conserved in fish and mice [51, 52].

It is important to note that while upregulation of MAPK-related genes and increased p38 protein levels coincided with autophagy activation, these observations alone are insufficient to establish that trehalose's antioxidant effect strictly depends on the “MAPK→autophagy” pathway [53]. For example, in zebrafish, the p38 MAPK pathway can regulate inflammation and apoptosis independently of autophagy to exert antioxidant effects [50]. For instance, researchers have demonstrated that the MAPK/ERK pathway promotes autophagy in hepatic oval cells to alleviate oxidative stress [19], whereas other researchers have reported that p38 MAPK protects cochlear hair cells from cisplatin damage by activating TFEB-dependent autophagy [48]. Notably, while these studies highlight the interconnection between MAPK signaling, autophagy, and oxidative stress regulation, the present work did not directly validate the causal relationship of “MAPK activation→autophagic enhancement→oxidative stress reduction”. Future studies could employ MAPK inhibitors (e.g., SB202190) or gene silencing techniques (e.g., RNAi) to block this pathway and determine whether trehalose's protective effects are attenuated, thereby strengthening the reliability of conclusions about this signaling axis (referencing [53]′s use of p38 inhibitors to validate pathway dependency in oxidative stress models).

## 5. Conclusion

In summary, the findings of this study indicate that dietary trehalose can increase endogenous trehalose levels in crabs exposed to AES. Elevation of trehalose may promote autophagy by potentially activating the MAPK signaling pathway, thereby alleviating the oxidative stress-induced injuries caused by AES in crabs. These results imply that trehalose holds the potential to serve as an effective feed additive for crabs, improving their capacity to withstand AES during culture and transport. These investigations will provide a more comprehensive and reliable scientific foundation for the application of trehalose in basic crustacean feed formulations, though further studies are needed to confirm the specific regulatory mechanisms involved.

## Figures and Tables

**Table 1:** Composition and percentage (%) of the control diet.

Ingredient	Content
Soybean meal	15.50
Peanut meal	8.00
Rapeseed meal	18.00
Cotton meal	7.00
Fish meal	7.00
Wheat flour	28.30
Yeast meal	2.00
Squid powder	2.00
Phosphatide	2.00
Fish oil	1.50
Pork lard	1.50
Mineral mix^a^	0.30
Vitamin mix^b^	1.20
Ca (H_2_PO_4_)_2_	1.00
Choline chloride	0.40
Dishulin	0.10
Bentonite	4.00
Salt	0.20
Total analyzed composition	100.00
Mousture	11.45
Crude protein	34.56
Crude lipid	8.34
Ash	9.15

^a^ Vitamin premix (per kg diet): vitamin A, 62500 IU; vitamin D_3_, 15,000 IU; vitamin E, 1.75 g; vitamin K_3_, 35.4 mg; vitamin B_1_, 100 mg; vitamin B_2_, 150 mg; vitamin B_6_, 150 mg; vitamin B_12_, 0.2 mg; biotin, 4 mg; D, calcium pantothenate; 250 mg; folic acid, 25 mg; nicotinamide, 300 mg; vitamin C, 700 mg. -calcium pantothenate, 250 mg; folic acid, 25 mg; nicotinamide, 300 mg; vitamin C, 700 mg.

^b^ Mineral premix (per kg diet): FeSO_4_·H_2_O, 200 mg; CuSO_4_·5H_2_O, 96 mg; ZnSO_4_·H_2_O, 360 mg; MnSO_4_·H_2_O, 120 mg; MgSO_4_·H_2_O, 240 mg; KH_2_PO_4_, 4.2 g; NaH_2_PO_4_, 0.5 g; KI, 5.4 mg; CoCl_2_·6H_2_O, 2.1 mg; Na_2_SeO_3_, 3 mg.

**Table 2:** Primer sequences for RT-qPCR and PCR.

Prime name	Sequence(5′-3′)	Gene accession number
Trehalose metabolism genes		
TPS-F	GGACCTTCATTACTCGTTTTCACA	LOC126982678
TPS-R	CATCAACAAGGAAAGCATAGCA	—
TREH-F	TGTCCAACCTTCACCCCATA	LOC126987726
TREH-R	GGCACTCCACCAATGTAATCTA	—
TRET-F	GTGTTCGGTTTGCTTTTGTCTG	LOC126981497
TRET-R	GTCACGCCGTCGCATAATCT	—
Autophagy genes		
ATG4B-F	ACACCACTGTTCCTTTGCC	LOC127003469
ATG4B-R	GCGTCTTCTCCGAGTCTATCA	—
ATG7-F	GCAACCGAAGCAAACAAGA	LOC126987558
ATG7-R	AACGCTGGGAAGGCAAACC	—
ATG13-F	GCAGGGTTCGGACTGGTTCA	LOC126982362
ATG13-R	GAGGGTGTCATTGGTTGGTGT	—
Beclin1-F	TTGTATGGGTCTGGAGGTTTC	LOC126983051
Beclin1-R	GTTGATGCGATAAGGAAAGC	—
MAPK pathway-related genes		
p38-F	GTTTAGATGTTCTGGGTGAAGGAG	LOC126994253
p38-R	TTCGTGACGGAAGTAGTGGG	—
JNK-F	ATTACGGTGATGAGGGTGAA	LOC127004423
JNK-R	GTCGCAGTAGAGGCAGGATG	—
ERK-F	AGCCCAACGCCCACATAGA	LOC126980500
ERK-R	CCCGAGGATACAGCCCAAAC	—
18S-F	TCCAGTTCGCAGCTTCTTCTT	LOC126996877
18S-R	AACATCTAAGGGCATCACAGA	—

## Data Availability

The data are available with the corresponding author of this publication upon reasonable request.
